# Learning from Alfred Wegener’s pioneering field observations in West Greenland after a century of climate change

**DOI:** 10.1038/s41598-023-33225-9

**Published:** 2023-05-23

**Authors:** J. Abermann, B. Vandecrux, S. Scher, K. Löffler, F. Schalamon, A. Trügler, R. Fausto, W. Schöner

**Affiliations:** 1 Department of Geography and Regional Sciences, University of Graz, Heinrichstraße 36, 8010 Graz, Austria; 2 Know-Center, Research Center for Trustworthy AI and Data, Sandgasse 36, 8010 Graz, Austria; 3 Institute of Interactive Systems and Data Science, Graz University of Technology, Sandgasse 36/3, 8010 Graz, Austria; 4 Department of Glaciology and Climate, Geological Survey of Denmark and Greenland (GEUS), Øster Voldgade 10, 1350 Copenhagen, Denmark

**Keywords:** Climate change, Cryospheric science

## Abstract

The cryosphere in Greenland is currently undergoing strong changes. While remote sensing improves our understanding of spatial and temporal changes across scales, particularly our knowledge of conditions during the pre-satellite era is fragmented. Therefore, high-quality field data from that period can be particularly valuable to better understand changes of the cryosphere in Greenland at climate time scales. At Graz University, the last work-place of Alfred Wegener we have access to the extensive expedition results from their epic 1929–1931 expedition to Greenland. The expedition coincides with the warmest phase of the Arctic early twentieth century warm period. We present an overview of the main findings of the Wegener expedition archive and set it into context with further monitoring activities that occurred since, as well as the results from reanalysis products and satellite imagery. We find that firn temperatures have increased significantly, while snow and firn densities and have remained similar or decreased since. Local conditions at the Qaamarujup Sermia have changed strongly, with a reduction in length of more than 2 km, in thickness by up to 120 m and a rise in terminus position of approximately 300 m. The elevation of the snow line of the years 1929 and 1930 was similar to the one from the extreme years 2012 and 2019. Compared to the satellite era, we find that during the time of the Wegener expedition fjord ice extent was smaller in early spring and larger in late spring. We demonstrate that a well-documented snapshot of archival data can provide a local and regional context for contemporary climate change and that it can serve as the basis for process-based studies on the atmospheric drivers of glacier changes.

## Introduction

The unstable years between the two great wars of the twentieth century coincide with the most productive period of an outstanding scientist of recent times, Alfred Wegener. While he became famous for his work in the field of geology, his core-interest and the topic following through his entire life has been polar meteorology. After two important expeditions to Greenland as a very young man, he took a long break from polar science, not least due to duties as an officer in WWI^1^ and unstable research contracts^2^. The scientific liberty that he gained with his late professional appointment in 1924 as a professor of meteorology and geophysics at Graz University eventually liberated him from either too heavy teaching duties or administration and let him focus on the 1930–1931 expedition to Greenland, that was to become his last one.

The motivation of this endeavor was to learn more about weather and climate in Greenland as it had become obvious that this eventually impacts the climate in Europe. Furthermore, basic characteristics such as the thickness and properties of firn and ice were little known at that time. In order to capture an overview that entails the atmospheric dynamics and the geophysical fundamentals of Greenland, the idea was to perform glacio-meteorological measurements across the entire ice sheet, from West to East. The choice of the location of the transect stemmed from practical considerations such as access onto the ice (from both coasts), available manpower from local communities and was in addition based on the knowledge Wegener gained from his earlier crossing together with Koch^3^.

It was challenging but finally successful to raise funding mainly from the ‘Notgemeinschaft der Deutschen Wissenschaft’ (which would later become the German Research Foundation). Costs were estimated based on the experience of the Koch-Wegener expedition. The strong inflation of the years between the great wars made it difficult to budget reliably. In total, the amount raised was 280.000 Reichsmark^4^, which would correspond to roughly 1 million euros converted to the purchasing power of 2021^5^. The funding raised was substantial and surely made the Wegener expedition the largest funded project from public funds in Germany during those years. It can only be speculated what the main motivation was—be it strategy in terms of weather prediction or in parts also nationalism and the quest to write one more of the heroic stories of that time. While Wegener himself is not assumed to have been particularly German nationalistic^1^, the latter narrative has been exploited later in a Nazi perspective with the Wegener expedition serving as inspiration for the movie ‘S.O.S. Eisberg’^6^.

The large amount of data collected by Wegener’s 1930–1931 expedition has seen little use in science ever since. Only the work done at Eismitte (the measurement site in the middle of the ice sheet, EM) received broader attention in later studies^7^. It is also at that site where Ernst Sorge formulated what would be henceforth called *Sorge’s Law*^8^, which states that under a constant climate, the density of firn in a certain depth remains unchanged. Probably due to a combination of reasons (the tragic death of Wegener during the expedition in November 1930 and hence to the fact that he himself was not able to analyze the measurements collected; conflicts resulting among the expedition members involving Alfred Wegener’s elder brother Kurt^4,9^), a large part of these observations remains unused to date. Luckily, Kurt Wegener meticulously documented the expedition material and assembled it in a reproducible way^10^ in the framework of his appointment in Graz that he ‘inherited’ from his brother after his death. We do not know how many copies exist, however, at Graz University two complete sets are placed. The fact that the data has not been used extensively is particularly unfortunate as recently several noteworthy initiatives compiled historical glaciological and meteorological data in Greenland and the Arctic. Some mention the data^11^, some use them in lower resolution than available^7,12^, others do not use them at all^13^.

With this contribution, we aim to present the core findings of the 1930–1931 Wegener expedition in a modern perspective and provide a concise history of the area and volume changes of an outlet glacier in their study area (West Greenland) since the Little Ice Age (LIA). The data has been digitized at its original resolution and is openly available at a repository along with scanned original reports (see data availability). In addition to presenting the data in a modern perspective, we use it to document the early twentieth century warming period (ETCWP), validate reanalysis products, compare fjord ice conditions during the ETCWP to the satellite period, and document changes in snow densities over the past century.

## Study area

### Qaamarujup Sermia and West Station (WS)

**Figure 1:**
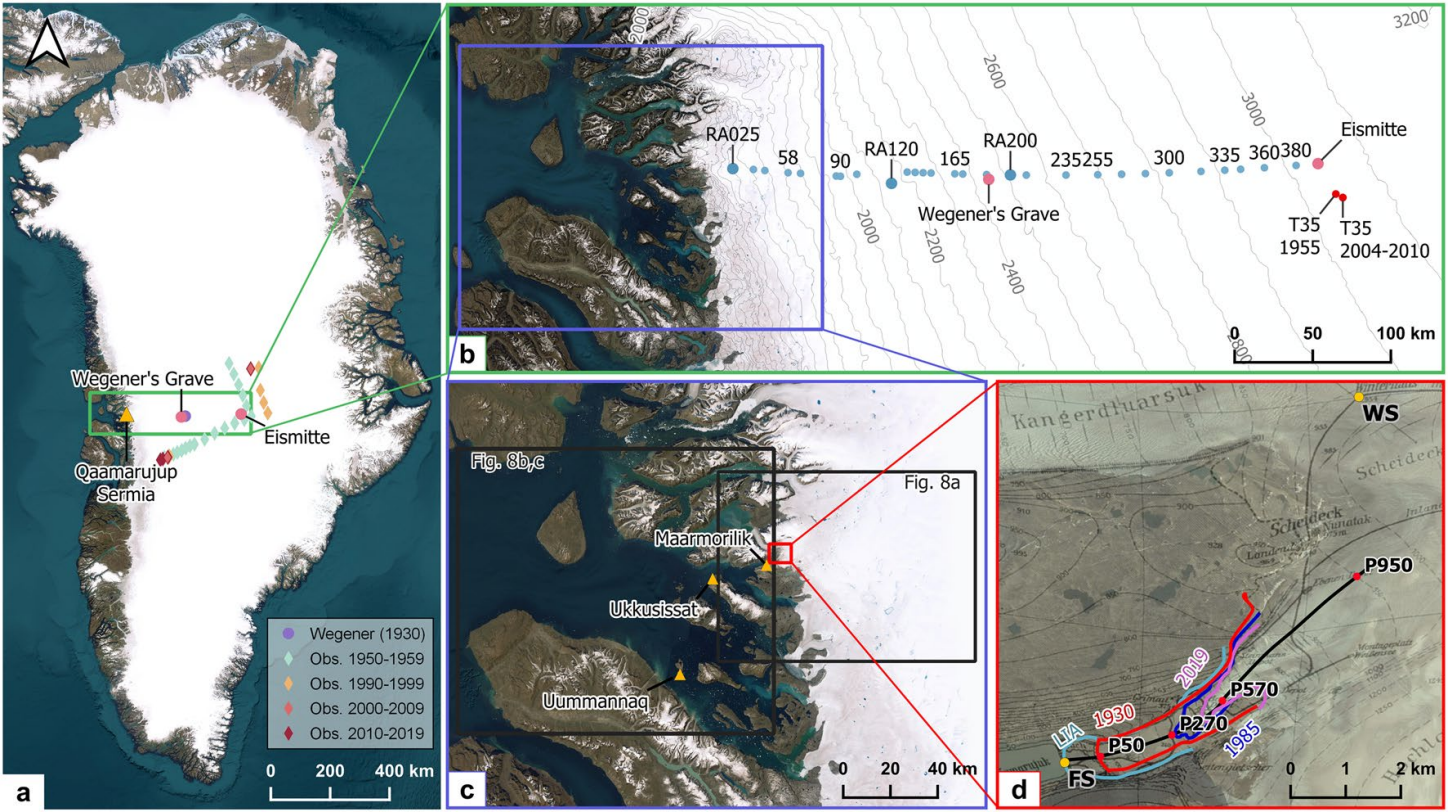
The study area in Greenland. (**a**) Overview of Greenland with some land-marks and the extended snow and firn measurement positions of Wegener’s expedition; (**b**) the transect between the coast and Eismitte (EM) including nearby firn density sites from later expeditions; (**c**) the extent near the coast with some settlements described in the study as well as the extent for Fig. 8; (**d**) the Qaamarujup Sermia with glacier margins of different points in time (coloured lines), a longitudinal profile (black line) and the weather stations Fjord station (FS) and West Station (WS) as well as the approximate location of the ablation stakes (red dots). The satellite imagery shown in the figure stems from Google Satellite imagery as the base layer from QGIS. All Figures were created in QGIS https://www.qgis.org/de/site/.

Access to the ice sheet and large parts of the glacio-climatological studies were carried out at the bottom of Qaamarujup fjord in West Greenland at 71°08′N, 51°10′W (Fig. 1a–d). The closest permanent settlement is Ukkusissat, approximately 25 km west of Qaamarujup fjord. A land-terminating outlet glacier (Qaamarujup Sermia) of the Greenland Ice Sheet lies at the easternmost end of the fjord. The glacier almost reached the coast during the Little Ice Age (LIA) based on moraine deposits, but since then underwent a significant retreat (Fig. 1d). The study area has had an interesting history in the past century, experiencing various types of human activity. The expedition organized by Alfred Wegener was the first visit of mainland Europeans^14^; however, it is unclear whether (and not to be excluded that) any Norse activity reached to this spot before that. The expedition was a major logistical achievement and occurred between 1929 (reconnaissance expedition) and 1930–1931 (operational). From the basecamp (close to the weather station‚ fjord station’ (FS) at 71°08′30″N 51°13′20″W) near the coast, several atmospheric and glaciological measurements have been done along the outlet glacier (Fig. 1d). Furthermore, a temporary research station has been built on the ice in the vicinity that was called ‘Weststation’ (WS) at 71°12′22″N 51°5′40″W. Around 20 scientists and several dozen logistical helpers from nearby villages were involved. After the Wegener expedition, the neighboring valley soon got used in an industrial setting through a minor marble quarry in 1933 and later on a Zn, Pb and Ag mine, which led to the foundation of the settlement of Maarmorilik^15^. Between 1973 and its closure in 1990, nearly 12 million metric tons of ore were extracted from the mine. In the last decade there were some initiatives to reopen the exploration at that site^16^ but to our knowledge no concrete plans are under way to reopen.

### The transect over the ice sheet to Eismitte

Apart from the work in the vicinity of the base camp at the bottom of Qaamarujup fjord at FS and WS, work was carried out at a transect from the margin toward the ice divide, where station EM was established at approximately 71°11′N, 39°56′W, 3010 m above sea level (a.s.l.). EM was supplied through Qaamarujup fjord over a transect that was traversed several times by different expedition members along which also scientific experiments and measurements were taken. The reports from the historical expedition refer to the positioning along the transect as km ‘Randabstand’, referring to ‘distance to the margin’. We keep this nomenclature for the positions along the transect by introducing ‘randabstand’ (RA) along with a number that can be understood as an approximate distance to the ice margin but emphasize that the distance to the ice margin is naturally changing with glacier changes. RA should thus be understood as a placename instead of a Euclidean distance. RA200 would hence refer to approx. 200 km from the margin as defined by the Wegener expedition. Figure 1b gives an overview of the transect where the expedition had taken place as well as a close-up of the area around the WS. Glacier extents for different years are shown (Fig. 1d).

## The climatological context

The twentieth century started with rather cool conditions until the 1920s, whereafter a rapid and noticeable warming occurred, peaking in the early 1930s^17^. A period of gradual cooling prevailed until the late 1980s^18^ followed by a period of well-constrained warming since the 1990s. This generalized temperature history of Greenland is rather uniform, with (North-)West Greenland being the area with the strongest warming^19,20^. The recent warming has been put into a larger spatio-temporal context^19–22^ and it has been concluded that the ETCP and the warm phase since the 1990s have been of a comparable magnitude. This is remarkable, as the atmospheric drivers have different origins: It has been postulated^17^ that the drivers for the ETCWP are a combination of internal natural climate variability in the North Atlantic and positive feedbacks that amplified the radiative and atmospheric forcing (e.g., through low volcanic activity, and growing greenhouse gas emissions), which is in line with a recent study^23^. The ETCWP needs to be put into a regional perspective^24^ and a connection to natural circulation variability as well as to sea surface temperature anomalies as possible mechanisms is established^25^. Changing circulation patterns are supported by increased levels of sulfate and black carbon concentrations in ice cores^26,27^. Radiation trends across the Arctic show that the ETCWP coincides with a phase of generally strong solar incident radiation^11^; the recent atmospheric warming in contrast is largely caused by anthropogenic emissions of greenhouse gases that lead to more emitted longwave radiation from the atmosphere^28^. Additional feedback processes such as oceanic warming and a decrease in sea-ice and snow cover lead to the Arctic warming up to four times as fast as the rest of the globe^29–31^.

Large-scale atmospheric forcing impacts Greenland’s climate, of which the North Atlantic Oscillation (NAO) and the Greenland Blocking Index (GBI) often are used as relevant indicators^32,33^. In terms of NAO, the study area in West Greenland is at an interesting boundary. There is a negative correlation between accumulation patterns and the NAO index for the ablation area of West Greenland, while it is neutral or slightly positive for the accumulation area of the transect between Qaamarujup and EM^32^. The ETCWP was generally characterized by a positive NAO and a shift from negative to positive GBI. Based on the NAO signal, during the ETCWP, below-average accumulation can be expected in the ablation zone and around average in the accumulation zone^32^.

The long air temperature record at the West Greenland town of Upernavik (less than 250 km from the Wegener’s expedition base camp) illustrates the temperature history in the region since 1784 episodically and continuously since 1870. We show air temperature anomalies together with the NAO index since 1900 in Fig. 3a,b. The period between 1870 and 1925 showed relatively cold temperatures with some years up to almost 4 °C below the 1981–2010 average. A steep rise was initiated in the early 1920s and peaked in the early 1930s just around the Wegener expedition (grey vertical line), after which some cooler decades came until the initiation of the recent warming.

## Data

The results, data and metadata of the expedition were published in seven books, categorized in the history of the expedition, seismic studies, glaciology, meteorology, geodesy, anthropology and zoology. Supplementary Table S1 gives an overview of data available and their position in the scanned expedition reports. All data including the scanned reports are available through a repository (see data availability).

## Results

In the following, we present the findings of our study, separated into changes in glacier morphology, melt rates and accumulation, meteorology, snow and firn temperatures and densities, end of summer snow line, and fjord ice conditions.

### Changes in glacier morphology

**Figure 2:**
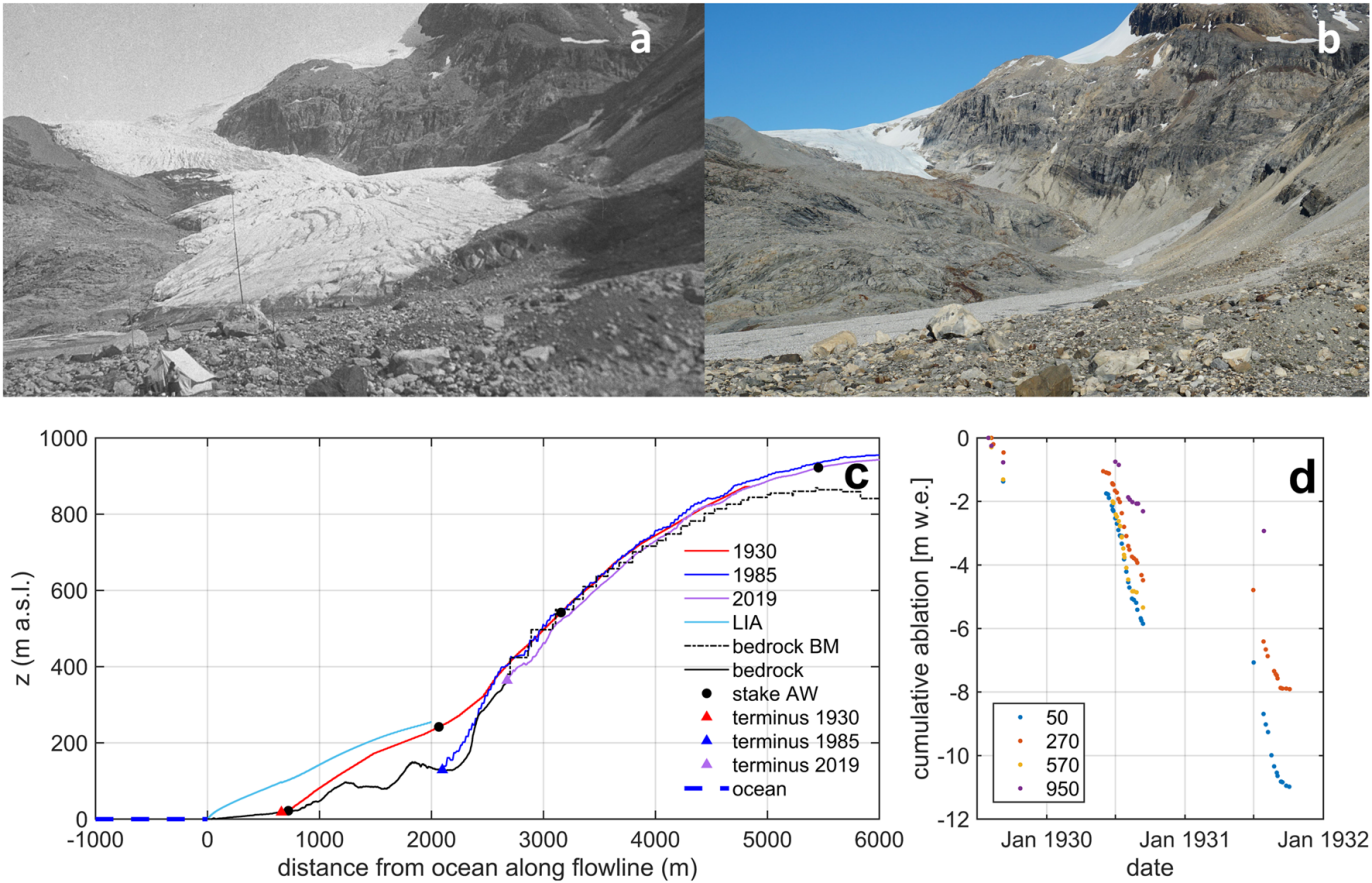
Glacier changes: Comparison of Qaamarujup Sermia in (**a**) 1930 and (**b**) 2022 taken from the same position near the shore. Note the boulders in the foreground that verify the same location. (**c**) Cross-section of Qaamarujup Sermia showing glacier surfaces during the LIA as well as in 1930, 1985 and 2019, and including the modelled bedrock topography by Bedmachine (BM)^34^. The positions of the ablation stakes are shown as black dots. (**d**) Ablation rates quantified by Wegener^35^ for the period 1929–1931 along a transect at elevations of 50, 270, 570 and 950 m a.s.l.

Although the Little Ice age (LIA) moraine is clearly visible at the bottom of the fjord, the exact timing of the maximum extent can only be speculated but it was already clear during Wegener’s expedition that ice had reduced significantly since the LIA maximum. This is in line with observations available to them when comparing their contemporary extents with the famous Drygalski expedition’s results^36^ at several outlet glaciers in the area around Uummannaq. It is reported explicitly that the glacier experienced a recession already in 1929 which is argued with the morphology of the glacier front^14^. Terminal retreat of 3 m was measured during summer (July to September) 1930 and another 15 m until August 1931. Without further moraine dating it is thus impossible to deduce an accurate date of the LIA maximum. It was clearly before 1929 and likely before or around 1891 where several glaciers in the vicinity were visited and stationary or retreating conditions were observed^36^, which is in line with a recent review on LIA extents in Greenland^37^. Continuous glacier recession has taken place since the Wegener expedition that led to strongest volume losses below 200 m a.s.l. (up to 120 m thickness loss in places) and a frontal recession of around 2 km horizontal distance (Figs. 1d and 2a–c), which would mean an average length change rate in the order of 20 m per year. This exceeds the values reported by the Wegener expedition^14^, which is surprising given the fact that the glacier tongue is now at higher elevations. However, it seems that the thinning of the ice and the associated downwasting accelerates margin retreat rates and outweighs the recession into higher elevations.

Qaamarujup Sermia today is rather narrow and does not exceed an ice thickness of a few dozens of meters according to model results^34^. Ice thickness increases markedly from 800 m a.s.l. upward as the bedrock overdeepens above the elevation of WS. Future thickness changes are thus expected to strongly impact the remaining thin glacier tongue of the outlet glacier at a high rate; once this is gone, rates of changes may reduce.

### Melt rates and accumulation

From the results of the Wegener expedition, we have a good indication of sub-seasonal melt rates at four stake locations in the ablation zone between 50 and 950 m a.s.l. (ST50, ST270, ST570 and ST950). In Fig. 2d we show the cumulative ablation based on the expedition reports^35^. Ablation in the season 1929/1930 amounted to 4.2 m w.e. at 50 m a.s.l. and 1.6 m w.e. at 950 m a.s.l. (Fig. 2d). This leads to a vertical mass balance gradient of about − 280 mm w.e./100 m. The season 1929–1930 showed less mass loss than the season 1930–1931 (about 1 m w.e. more ablation in 50 m a.s.l.), however, average vertical mass balance gradients were similar.

**Figure 3:**
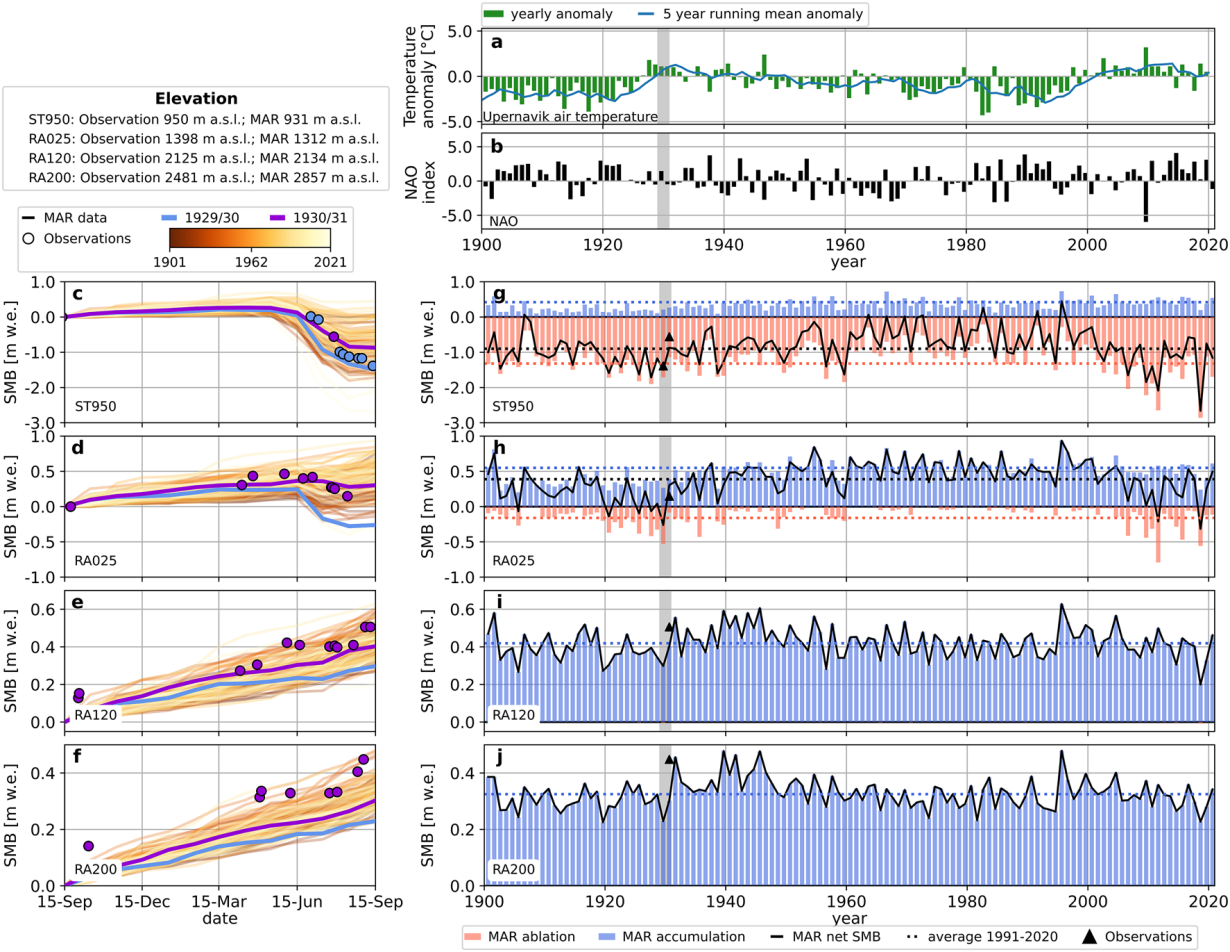
Climate and mass balance since 1900: (**a**) annual air temperature anomalies relative to the 1991–2020 average at Upernavik weather station^39^ (green bars) with a 5-year running mean (blue line). The warming during the ETCWP stands clearly out. The timing of the Wegener expedition is marked with a grey vertical bar. (**b**) The annual average of the NAO index^40,41^. (**c**–**f**) Annual cumulative surface mass balance as modelled with MARv3.5.2 and MARv3.12 (lines), where the line color indicates the year. Manual readings from the Wegener expedition are displayed as dots. The blue (purple) color refers to the expedition years 1929/1930 (and 1930/1931). (**g**–**j**) Annual accumulation (blue bars), ablation (red bars) and total surface mass balance (solid black lines) as modeled with MARv3.5.2 (1900–2010) and MARv3.12 (1950–2021). The observations from the Wegener expedition are shown as triangles. The dotted lines denote the average accumulation (blue), ablation (red) and net balance (black) for the period 1991–2020.

In Fig. 3c–j we assess the mass balance seasons of the Wegener expedition and their measured stake data in a long-term context by comparing gridded mass balance output from the Modèle Atmosphérique Régional (MAR) model^38^ over the past century. From 1900 to 1949 MARv3.5.2 (forcing grid: ERA20C; surface mass balance grid: 5 km) is used, between 1950 and 2010 the average of MARv3.5.2 and MARv3.12 (forcing grid: ERA5; surface mass balance grid: 1 km), and from 2010 onward MARv3.12. The Pearson correlation coefficient between both model versions is between 0.82 and 0.91 for the different locations during the overlapping period (1950–2010). The conversion from stake measurements from the Wegener expedition to seasonal (net) mass balance is done using measured snow densities at the respective locations. They are available for the transect from WS to EM. For ST950, net mass balance change refers to glacier ice with an assumed density of 900 kg/m^3^. Each time series represents one cumulative mass balance season (based on monthly data starting in September) between 1900 and 2021.

MAR captures the order of magnitude of both accumulation and ablation at the respective sites reasonably well. It is interesting that the accumulation of 1930/31 is extraordinarily low for ST950 and RA025 and above average further up. While measurements indicate stronger melt in the season 1930/31 than 1929/30 for the lower stakes (Fig. 2d), ST950 and the locations higher up along the transect to EM indicate more mass gain in 1930/31 than in 1929/30. A direct comparison between MAR output and ST50, ST270 and ST760 did not give reasonable results as vertical gradients of ablation are strong and MAR does not resolve the topography sufficiently there.

Figure 3g–j shows annual accumulation, ablation and net balance defined as cumulative positive, negative and all monthly values starting in September each year. Comparing the years immediately prior to the Wegener Expedition with the climatological average (1991–2020) indicates lower than average accumulation at all locations and particularly strong ablation anomalies in the lower part of the Greenland Ice Sheet (ST950 and RA025). The measured net balances by the Wegener expedition correspond well with the modelled mass balance from MAR. The ETCWP stands out clearly in air temperature anomaly records from Upernavik (Fig. 3a) and coincides with the transition from largely positive NAO to neutral conditions (Fig. 3b).

### Meteorology

**Figure 4:**
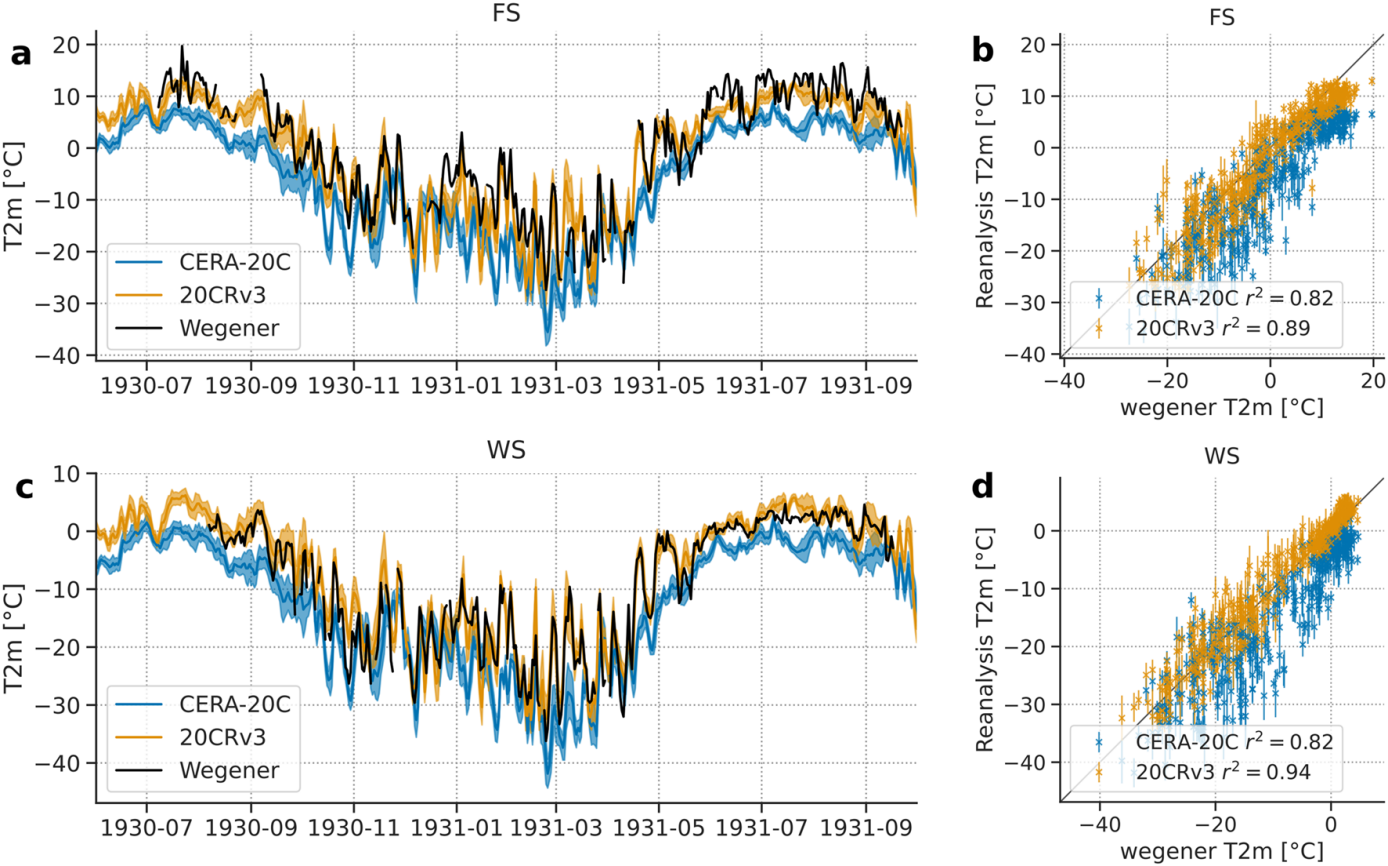
Daily mean air temperature (black) measured during the Wegener expedition in comparison to two reanalysis products (CERA-20C: blue and 20CRv3: orange), linearly interpolated, using height-correction with a constant lapse rate of − 6.5 K/km. (**a**,**c**) Time series of daily mean air temperature for FS (**a**) and WS (**b**); shading indicates uncertainty of the reanalysis (standard deviation of the individual ensembles) (**b**,**d**) scatterplot of daily mean air temperatures (measured vs. reanalysis), error bars show standard deviaton of the reanalysis ensembles.

Meteorological data is available for FS and for WS for the period 1930 to 1931 (Fig. 4). Daily average temperatures range between approx. − 37 °C at WS and 20 °C at FS with a clear seasonal cycle. The summer months June to August show smaller temperature variability compared to the winter months at both sites. At WS, daily average temperatures just above 0 °C are present for June to August. An average vertical air temperature gradient of − 0.88 °C/100 m is found, which is weaker during spring (− 0.46 °C/100 m in March) and gets stronger with the onset of the melt season, reaching -1.07 °C/100 m in November.

We assess the potential of reanalysis data to reproduce this poorly constrained early instrumental period and compare the measurements at FS and WS with two twentieth century reanalysis products, namely ECMWFs CERA-20C^42^ and NCEPs 20CRv3^43^. Reanalysis assimilates measurements with a numerical forecast model, to obtain the best estimate of the atmosphere’s state at each timestep. Both CERA-20C and 20CRv3 only assimilate surface pressure measurements. They are, therefore, much less constrained by measurements than reanalysis products for the satellite era (e.g., ERA5), which assimilate a wide range of different measurements, both in-situ and from remote-sensing.

Both reanalysis products provide 2 m air temperature at 3 h intervals, on a 1 × 1° grid. We linearly interpolate those spatially to the locations of FS and WS. Additionally, we apply a height correction from the model orography to the altitude of the stations, using a constant lapse rate of − 6.5 K/km.

During the Wegener expedition, for FS, temperature was measured three times a day, at 0700, 1300 and 2000 UTC (in the reports at 0800, 1400 and 2100 CET), and they computed the daily mean as

$$T_{mean} = \frac{{\left( {2 \times T_{0700} + 2 \times T_{1300} + 5 \times T_{2000} } \right)}}{9}.$$

The reanalysis data is available at 0000, 0600, 1200, 1500, 1800 and 2100 UTC. We use the snapshots that are closest in time to the Wegener measurements, thus the snapshots at 0600, 1200 and 2100 UTC, and the same weights, leading to

$$T_{mean} = \frac{{\left( {2 \times T_{0600} + 2 \times T_{1200} + 5 \times T_{2100} } \right)}}{9}.$$

At WS temperature was recorded every other hour from which we computed the daily mean directly and compare this with in the daily average of the 3 h reanalysis data.

The results for comparison are shown in Fig. 4a,c as time series and in Fig. 4b,d as scatter plots including the Pearson correlation coefficient. Both reanalysis datasets capture the seasonal cycle well, but the variability on shorter timescales is not always captured correctly. 20CRv3 matches more closely than CERA-20C in that respect. CERA-20C shows a remarkable cold bias, which might be caused by the known issue that CERA20-C has poorly represented sea-ice—atmosphere coupling, which leads to an overestimation of sea-ice^42^. The measurements at FS show a stronger short-term variability in summer 1931 than at WS (2–4 times higher 10-day running standard deviation)—this has likely to do with the ice surface compensating small-scale variability by capping the near-surface layer to melt conditions and is not captured by any of the two reanalysis products. At both stations, the variability is higher in winter than in summer. Interestingly, the match between both reanalysis products and station data is better in winter than in summer.

**Figure 5:**
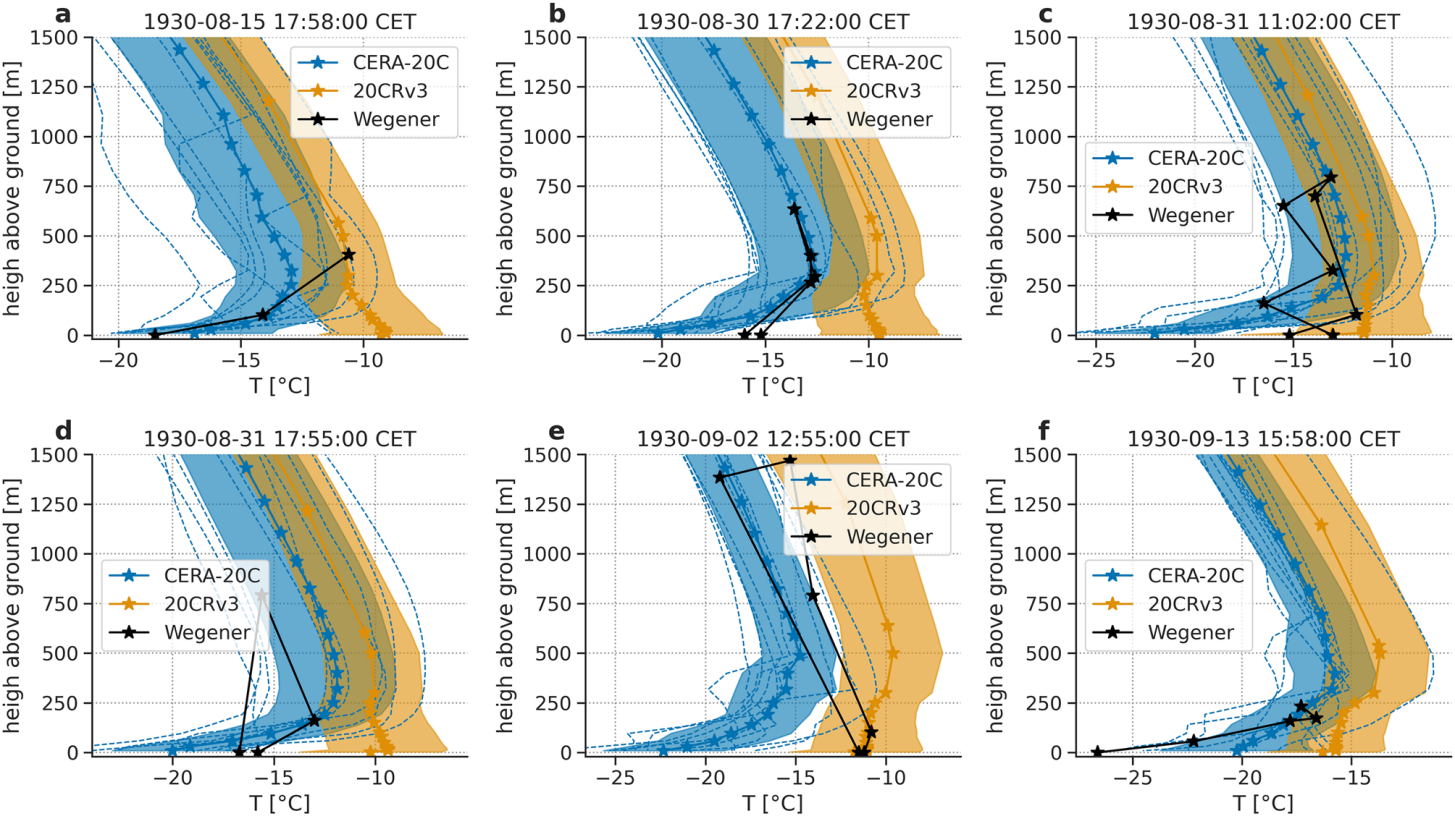
Vertical stratification: Air temperature profiles from kite ascents at EM measured by the Wegener expedition (black) compared to CERA-20C (blue) and 20CRv3 (orange). Shading shows ensemble standard deviation, dotted lines show individual members of CERA-20C. Date and time indicate the starting time of the ascent.

Additionally, we analyze vertical temperature profiles, which we compare to 6 ascents from a temperature sensor equipped kite made at EM. 20CRv3 provides air temperature data directly at defined heights above ground level (a.g.l.), between 12 m a.g.l. and 500 m a.g.l.. Above 500 m a.g.l., we use pressure level data of air temperature. The height at each timestep is derived from the geopotential of the pressure level. Additionally, we use 2 m air temperature data. CERA-20C does not provide data on height levels. Instead, we used model-level data for all heights, which we converted to height levels. Both CERA-20C and 20CRv3 provide measures of uncertainty, obtained from ensemble data assimilation (10 members for CERA-20C, 80 for 20CRv3). For both, we show the standard deviation of the ensemble in addition to the ensemble mean. The temperature profiles from kite ascents at EM are shown in Fig. 5 together with the vertical stratification from reanalysis products. CERA-20C shows strong inversions, in contrast to 20CRv3. In Fig. 5a,b, there is an inversion present in the measured data, which is absent or weak in Fig. 5c–e. Figure 5b–e include data both from the kite ascent (and descent), and thus gives an indication of uncertainty in the kite ascents. Regarding the comparison with reanalysis products, we note, that 20CRv3 does not reasonably reproduce inversions, while CERA-20C does. On the other hand, in Fig. 5e, CERA-20C does show an inversion, even though the measurements do not. Thus, for both reanalysis products, care must be taken if they are used in any context that requires accurate temperatures close to the ice sheet or the analysis of vertical stratification.

### Firn temperatures

**Figure 6:**
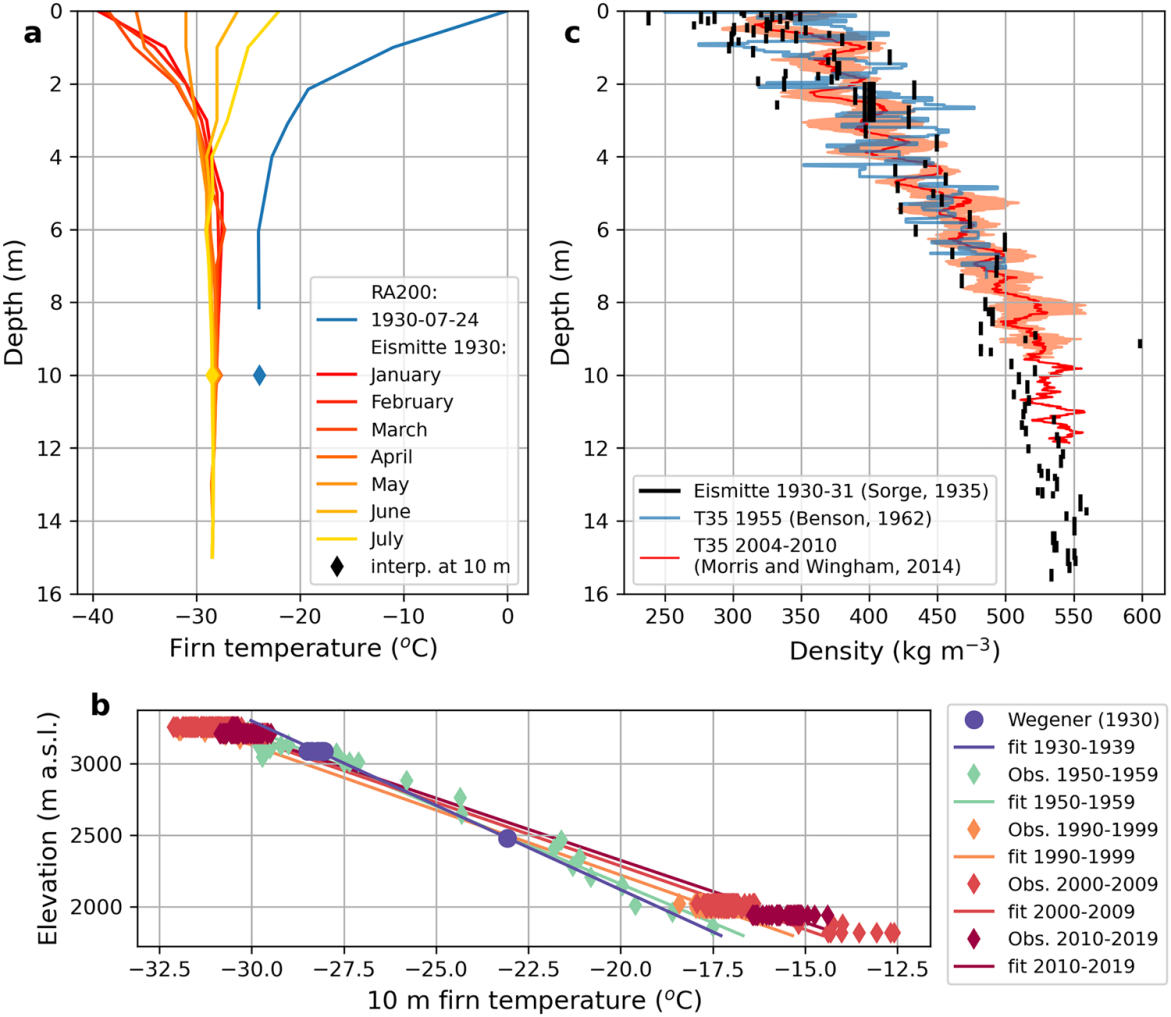
Firn properties: (**a**) Firn temperatures measured at RA200 (blue line) and EM (red to yellow) along with interpolated 10 m firn temperature (diamond). (**b**) 10 m firn temperature measurements from various observations and points in time together with a linear interpolation thereof. Wherever more data points for one site are shown, we display monthly averages. (**c**) Firn density profile at EM measured in 1930^44^ and the nearby site T35 measured in 1955^45^ and 2005–2010^46^. For the four profiles from Morris and Wingham^46^, their average (solid red line) and standard deviation (shaded area on both side of the solid line) are displayed.

Firn temperatures were observed by Wegener at RA200 on 24 July 1930 and by Sorge^44^ at EM from November 1930 to August 1931 (Fig. 6a). The 10 m firn temperature (T_10m_) is taken as a reference of long-term climate because in high elevations it mainly depends on the surface temperature and on the heat diffusion through snow and firn. At EM the T_10m_ was on average − 28.2 °C from January to July with a standard deviation below 0.2 °C. At RA200 a T_10m_ of − 23.9 °C can be interpolated from the measurements at 6.05 m and 8.25 m. We compare these T_10m_ values with T_10m_ observations from the 1950s^45,47,48^, the 1990s^7,49,50^, the 2000s^7,51,52^ and the 2010s^49,53^. All these observations are from within 330 km from EM and are selected upon the following availability criteria: (I) each decade contains at least two T_10m_ observations with (II) more than 500 m of elevation difference. The T_10m_ elevational gradients for each of these are: − 0.85 °C/100 m for 1930–1939, − 0.92 °C/100 m for 1950–1959, − 1.10 °C/100 m for 1990–1999, − 1.13 °C/100 m for 2000–2009, − 1.15 °C/100 m for 2010–2019 (Fig. 6b). This increase of the T_10m_ elevation gradient illustrates the firn warming in response to atmospheric warming that impacted lower elevations of the Greenland ice sheet to a stronger degree than areas higher up^54,55^. The T_10m_ elevational gradient was similar in the 1930s and in the 1950s and decreased to below − 1 °C/100 m by the 1990s. Although insufficient T_10m_ observations are available in 1960–1990 to document the exact timing of that change, weather stations and climate models show that the onset of the atmospheric warming took place in the mid-1990s^56^. The Wegener data gives an unprecedented historical perspective to this warming and shows that during the ETCWP firn was not as warm as we are measuring it today.

### Firn and snow densities

Firn density was measured at EM in a 15 m deep pit that was excavated between September 1930 and April 1931 (Fig. 6c). The reason for this long period is the fact that excavating 15 m of firn was very labor-intensive (in total more than 300 man-hours have been used to dig the pit). The density profile at EM is compared to later density profiles from the surroundings (Fig. 6c). EM is located 25 km away from the site T35 on the well-studied EGIG line (Expédition Glaciologique Internationale au Groenland). At T35, one density profile is available from 20 July 1955^45^ and four density profiles spanning from 2004 to 2010^46^ which were aggregated to their average for easier visualization. Remarkably, no significant shift in density can be seen at that site (Fig. 6c). This corroborates findings from earlier studies^57,58^ who also found that the high elevation areas of the ice sheet did not see a significant change of their firn density over the last decades.

**Figure 7:**
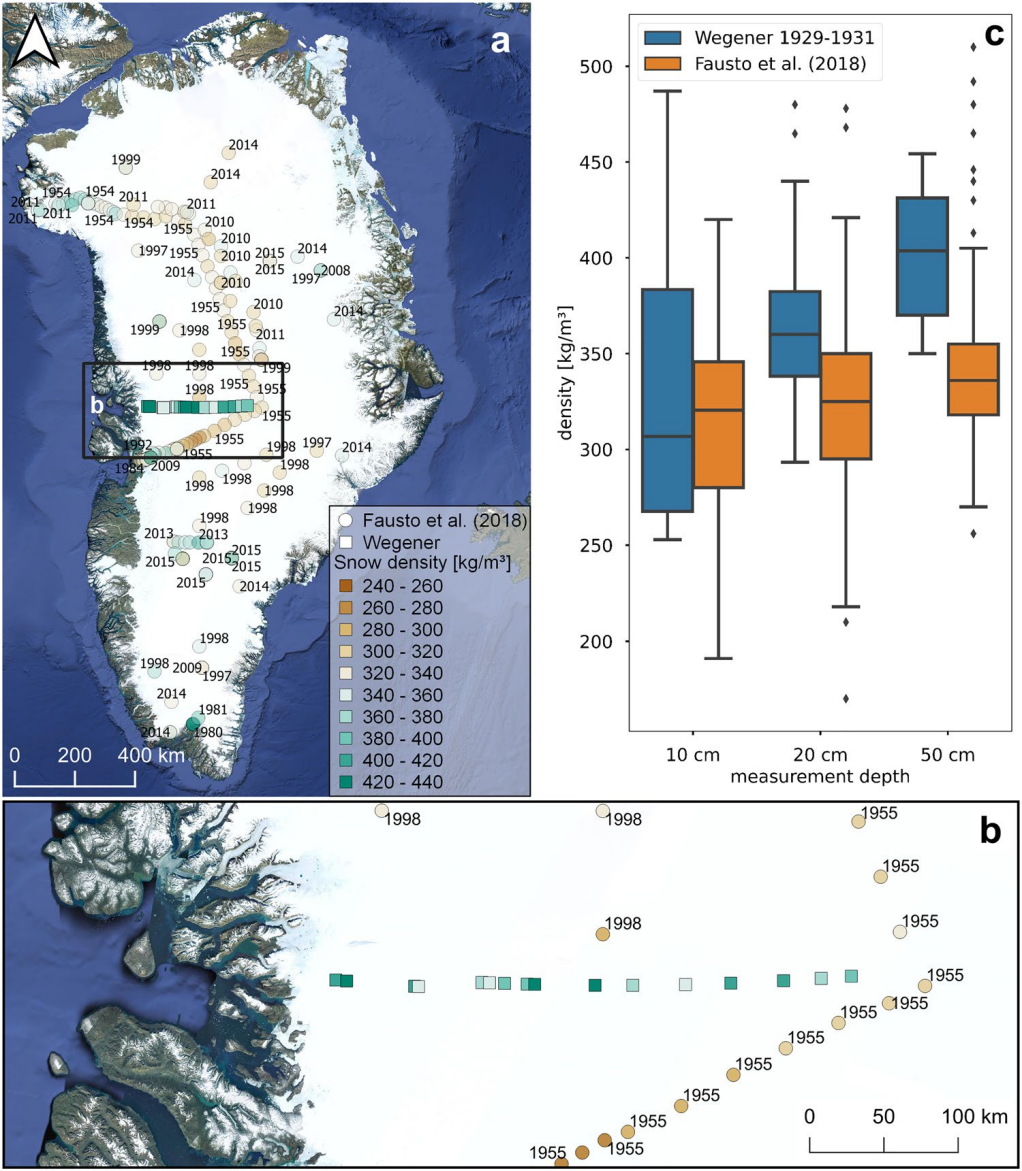
Snow densities: (**a**) The Fausto database (dots) includes all available snow density data and is here expanded with the Wegener pit data (squares). All data in from 50 cm below the surface is shown in the same colour signature. The Wegener data seems to point to systematically higher snow densities in depths of 20 cm and more, irrespective of their elevation. (**b**) As in (**a**) but with a zoom into the study area. (**c**) Snow density in 10, 20 and 50 cm below the surface comparing the Wegener data (blue bars) with the Fausto database (orange bars). The Wegener expedition recorded systematically higher snow densities. The satellite imagery shown in the figure stems from Google Satellite imagery as the base layer from QGIS. All Figures were created in QGIS 3.28 https://www.qgis.org/de/site/.

In addition to the deep firn pits, Wegener and his team measured snow density at 68 shallow snow pits between 16 July 1930 and 11 July 1931 along their transect to EM (Fig. 7a,b). Snow densities have been measured diligently and with a focus on maximizing accuracy^59^. A recent compilation of snow densities^13^ did not take the Wegener data into account. A comparison with their products that span a time-period of six decades shows generally lower densities than the Wegener expedition did (Fig. 7c). Differences are in the order of 50 kg/m^3^ which should be beyond the estimated uncertainty of manual measurements.

### End of summer snow line

**Figure 8:**
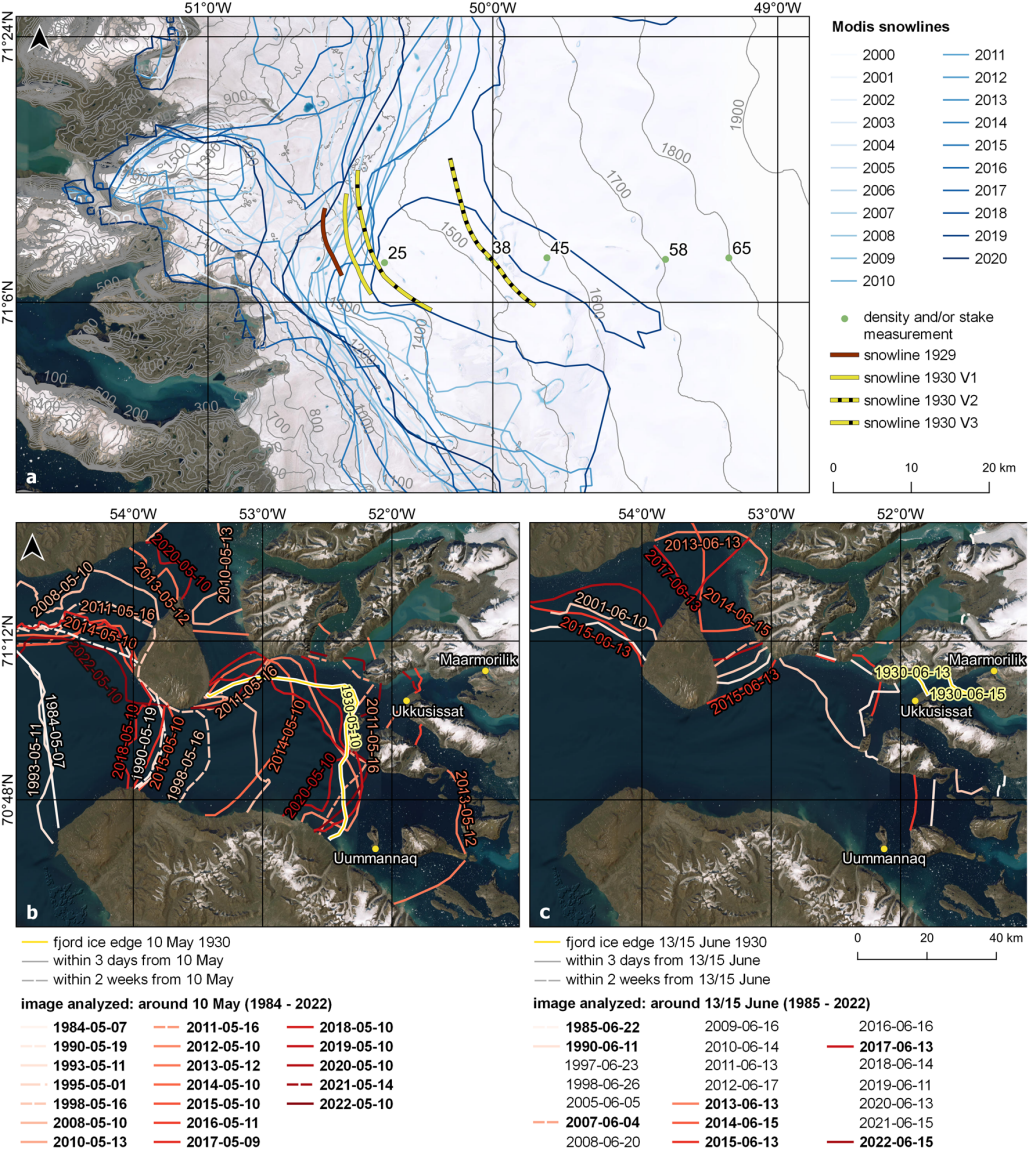
Snowlines and fjord ice cover: (**a**) Snowlines as derived from MODIS data^61^ compared with Wegener’s snowlines in 1929 and 1930 referring to different versions in their records. The snowline was higher than in 2012 and at a similar elevation as in 2019. (**b**) and (**c**) Ice extent based on the map of the expedition^60^ for (**b**) 10 May 1930 and (**c**) 13/15 June 1930 together with fjord ice extents derived from satellite images (Landsat 5/7/8, ASTER, Sentinel 2, MODIS Aqua). Satellite images within 3 days of the respective date are shown as solid lines, those within 2 weeks as dashed lines. The darker the red, the more recent the extent is. Bold font in the legend means that ice edge is displayed. Note, that years that appear in the legend but are not displayed indicate that ice has disappeared by the relevant date. The satellite imagery shown in the figure stems from Google Satellite imagery as the base layer from QGIS. All Figures were created in QGIS 3.28 https://www.qgis.org/de/site/.

To compare the end of summer snow line we compare various sources describing the snow line in the expedition reports. The snow line for 1929 was recorded on 4 Sep 1929^10,60^ (Fig. 8a). For 1930, the end of summer snow line appears in the reports at three instances^60^ (descriptive, relative to RA), we therefore add three versions (V1–V3) to the map, which should entail the possible extents. It is interesting to note, that regardless which evidence from the historical material we refer to, the snowline in the year 1930 was significantly higher than in the 2000–2020 average, in the same elevation or around 100 m lower than during 2019 and at a similar position as during the record melt year of 2012. For 1931 we have only indirect indication: RA25 had a net accumulation of 30–40 cm snow until 16 Sep 1931, and we know that Stake 950 (at Zeltplatz II) had significant ablation (net ablation: 1.95 m from 1930 to 1931). We can therefore only assume that the snow line was between 950 and RA25, likely closer to RA25. Since uncertainties are very high, we refrain from adding it to the map in Fig. 8a.

Uncertainties regarding the localization of the Wegener expedition stake readings exist, however, there are several reasons why we believe that the conclusions are robust: elevation has been derived by levelling which already in the 1930s was likely clearly in the accuracy range of better than ± 5 m also over long distances. The location of the transect from 25 km onward may be slightly misplaced and the map which it is based on is rather inaccurate. We expect a horizontal accuracy of the order of better than 5 km based on the map. This is consistent with elevation values that may certainly have changed in the accumulation area, too, but given a virtually unchanged surface elevation in 950 m a.s.l. at WS, we do not expect the elevations in 1300 m a.s.l. or higher to have changed by more than a few tens of meters. Hence, we can use the elevation as an independent assessment on whether the spatial positioning of the transect is realistic.

### Fjord ice conditions

One problem the Wegener expedition faced was the break-up of fjord ice that occurred particularly late in their view. This impeded the team to bring the expedition material to the bottom of Qaamarujup fjord, delayed the establishment of all on-glacier infrastructure and to some extent contributed to the tragic death of Wegener. It is interesting to assess whether their notion of a particularly late fjord ice break-up is true and how this would hold with modern conditions. The latter we can assess using remote-sensing data from the satellite era. From the map of the expedition material^60^ we digitized the approximate location of the ice edge on 10 May 1930 and from 13 and 15 June 1930 and show it in Fig. 8b,c. We then went through the archive of optical remote sensing imagery using the GEEDIT tool^62^ and digitized the ice edge, wherever applicable (bold font in legend). For images analyzed where no fjord ice was present, we show the date in the legend (regular font in legend). Based on this we could find the one closest to 10 May (Fig. 8b) and 13 June or 15 June (Fig. 8c) for the respective year. The fjord ice edge can vary widely, and it seems that the ice edge in 1930 had a rather small extent compared to other years for the early part of spring (e.g., 1995, 1998, 2008, 2018; Fig. 8b). While some years (e.g., 1993 or 1984; Fig. 8b) show an ice extent reaching more than 50 km further towards the open ocean, only few years in the past showed similarly low sea-ice cover around 10 May. For Mid-June we show, that fjord ice conditions were comparably large (given that for 13 years in the satellite era no fjord ice had been present anymore in June and only in 4 years was it larger than 1930; Fig. 8c).

Compared to recent decades, the ice extent during the Wegener expedition was thus comparably small in May and comparably large in Mid-June. What remains unclear at this stage is how far the conditions during the Wegener expedition deviated from the average of their time. According to the diaries and descriptions it seems that Wegener assumed ice cover to be abnormally large and long lasting, likely the only reference being oral narrative from the locals accommodating the waiting crew.

## Discussion

What we present above is an attempt to make unique glacio-meteorological data from a climatologically interesting period visible. Such measurements are particularly sparse in Greenland despite its heterogeneity, size, and importance for the global climate. This is true today, but even more so when we look back a century.

Simply digitizing old data and making it available can be a steppingstone for science. However, it is the contextualization with other monitoring activities that adds value to the community. We achieve this by comparing data from the Wegener expedition with records thereafter as well as with model output. The careful assessment of metadata and methodological accuracies is crucial when interpreting contrasts and differences. The snow density differences found are a good example for that. The historical material^59^ emphasizes on the description of the density measurements and gives the impression that high accuracy is achieved. The fact that no snow density changes are recorded for EM, yet, the more coastal and near-surface sites do show a significant difference, gives credence to deviations beyond measurement uncertainties.

Along those lines, also the good match between reanalysis products of 2 m air temperature and the historical measurements (that are not constrained or assimilated with those measurements) do support the fact that the data of the Wegener expedition are of reliable accuracy. Despite the impressive length of the expedition covering two melt seasons, the data gained through it do not allow an assessment of climate trends as this is way below natural variability. However, the combination of measurement data with twentieth century reanalysis data and/or model output, using both classical statistical as well as newly developed methods of machine learning has the potential of aiding our understanding of climate variability and trends, particularly when constrained with reliable validation data.

**Figure 9:**
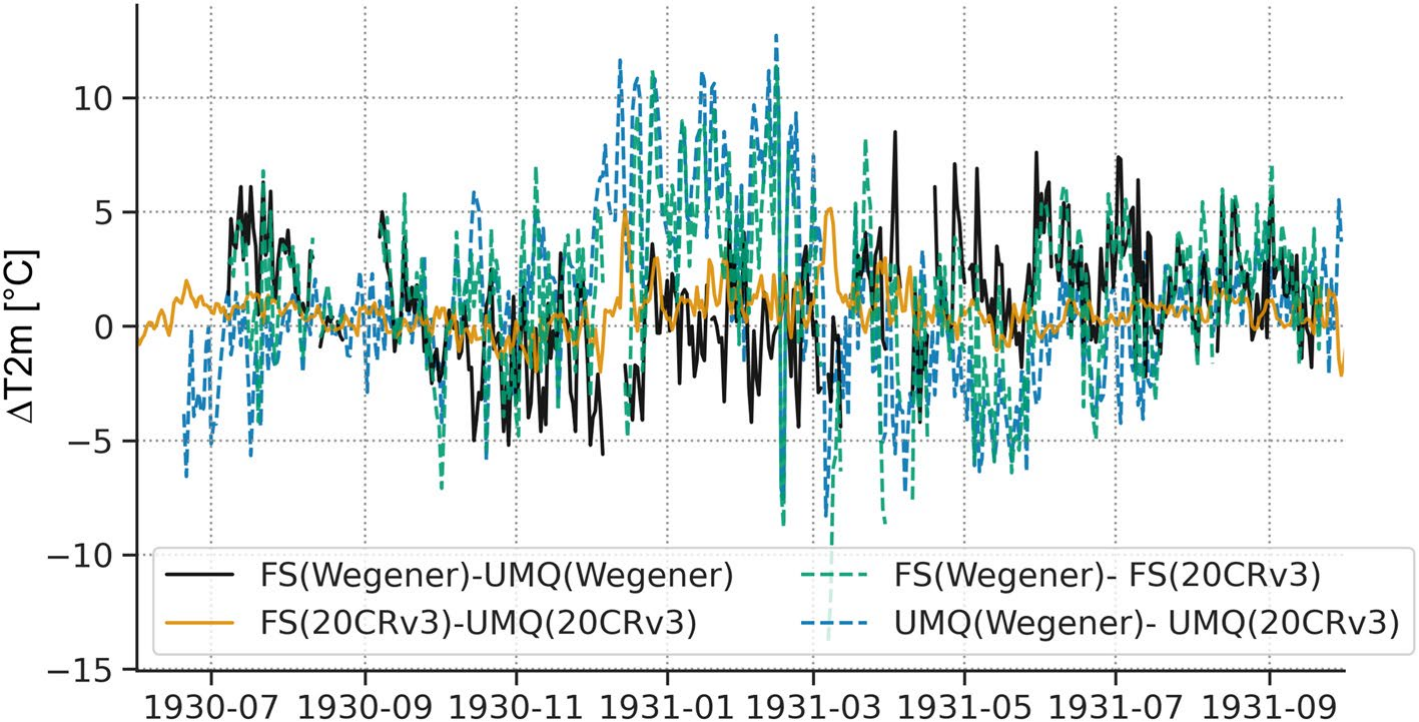
Air temperature difference between FS und UMQ based on observations (black solid line), the 20CRv3 reanalysis product (orange solid line) and the respective bias at each grid point (dashed lines).

Additionally, the locally observed spatial atmospheric variability can be compared with the respective model output. Given the good match between observations and reanalysis, a relevant question to ask is whether observed differences of atmospheric variables exceed the ones modelled by reanalysis products. Figure 9 shows the air temperature differences (∆T2m) between the best matching reanalysis product and the observations for the data points FS and UMQ, respectively (dashed lines) and in contrast temperature differences between FS and UMQ for both the observations (black) and reanalysis-data (orange). Figure 9 shows that there is a seasonal cycle in the deviations between observations and reanalysis data (positive difference in winter, negative in spring; dashed lines). Furthermore, we find that the air temperature differences between model and observation are rather similar for two locations 60 km apart (dashed lines). We also use this discrepancy to state that the spatial variability captured by observations (i.e., difference between FS and UMQ weather station; black solid line) is often more than twice the difference of the respective reanalysis product’s gridpoint (solid orange line). While the reanalysis products capture the temporal variability very well on a regional scale (Fig. 4), the local conditions may be captured with considerable differences (several °C on diurnal averages), which is a relevant magnitude when it comes to accurately reproducing melt rates.

## Conclusions and outlook

With the treasure trove of a well-documented snapshot of an interesting climatological phase, we show that we can assess questions regarding unprecedentedness and extraordinary conditions. We find for instance, that the fjord ice extent of the early 1930s was rather limited in May 1930 compared to contemporary conditions, while the mid-June extent was extraordinarily large. When it comes to the position of the snow line, we find, that despite large uncertainties, both 1929 and 1930 showed snowlines at the level of or higher than what we observed in recent years. This is even true for the extreme summers of 2012 and 2019. Large parts of the Wegener work have been digitized for this contribution and the entire data and metadata has been made available. Our analysis triggered a reconnaissance work that we implemented in 2022 and will run for the coming years. Having pinpointed the exact locations of visionary measurements from the 1930s we will be collecting both ablation and micrometeorological data there almost a century later. Due to the retreat of the glacier, the conditions under which the measurements are made today are fundamentally different to those in the 1930s—in parts what had been on-glacier ablation measurements then are locations where bare bedrock is exposed today. To make a connection possible, we make use of our thorough knowledge of the strong geometrical changes and apply innovative measurement techniques and a modernized and expanded monitoring at the Qaamarujup Sermia, inspired by the Wegener expedition.

## Supplementary Information

Supplementary Information.

## Data Availability

Scans of the original books from the Wegener Expedition, as well as the subset digitized for this study, are made available both on the projects GitHub repository (https://github.com/weg-re/wegener-expedition-data) and on Zenodo under 10.5281/zenodo.7890790. CERA20C can be downloaded from ECMWFs MARS archive (https://www.ecmwf.int/en/forecasts/datasets/reanalysis-datasets/cera-20c). NCEP 20CRv3 can be downloaded from NOAA (https://www.psl.noaa.gov/data/20thC_Rean/). The satellite imagery used can be accessed through the GEEdit Tool https://liverpoolgee.wordpress.com/geedit-geedit-reviewer/.

## Abbreviations

CERA: ECMWF reanalysis product
EM: Eismitte (central ice station)
ETCWP: Early 20th century warming period
FS: Fjord station
GBI: Greenland Blocking Index
LIA: Little ice age
MAR: Modèle Atmosphérique Régional
NAO: North Atlantic Oscillation
NCEP: NCAR reanalysis product
RA: Randabstand
WS: West Station
