# Supplementary material for: Learning from Alfred Wegener’s pioneering field observations in West Greenland after a century of climate change
Source: Sci Rep. 2023 May 23;13:7583. doi: 10.1038/s41598-023-33225-9 (PMC10205745; doi:10.1038/s41598-023-33225-9)
Supplement: Supplementary file 1 — Supplementary Information. [file 41598_2023_33225_MOESM1_ESM.pdf]

Summary of available data from the Wegener expedition 1929/30/31

The results and datasets of the expedition were published in 7 books along with detailed descriptions of the methods used. This is an overview of the available data and where to find them in the books. It is important to note that a lot of details are within the text describing the tables and figures, so it is highly recommended to read the whole chapter. All books are available as pdf, additional, some datasets were digitalized as .csv files – they are marked (*). Descriptions/Chapter are indicated by (C), tables by (T) and figures by (F).

Page	What	Details	Period	Resolution	Comments
Page in volume	Discriptive keywords	More details about what the data	In which time period the data is available	In which resolution (temporal/spatial) the data is available	Additional comments about the data, e.g- (C)/(T)/(F); (*)

Volume 1 History of the expedition

The scope of this volume is the history, technical details, and general information about the planning, executing and aftermath of the expedition.

Page	What	Details	Period	Resolution	Comments
1	Plan and execution of the expedition	An introduction to the expedition - Preconditions, planning and execution; description about transportation, equipment, tasks, participants, climate, area etc			(C)
60	Weststation	Description of the house functioning as the Weststation			(C)
62	Measurements Winterhaus	Temperature/wind outside compared to the temperatures inside the house			(T)
64	Transportation	Thoughts about the transportation issue during the expedition			(C)
77	Propeller sledge	Functionality and usage of propeller sledges			(C)
103	Radio communication	Setup of the radio communication, disruptions and list of successful communication			(C)
140	Cosmic radiation at sea level	Radiation measurements during the ship travel to Greenland			(T)
148	Cosmic radiation on the ice sheet	Radiation at different altitudes on the ice			(T)
151	Barometric altitude curve	Figure with barometric curve from Scheideck to approx. 250km; Altitude vs distance from ice margin			(F)

155	Mass balance	Stake measurements on the transect towards Eismitte, approx. every 20 km	August 1930 - September 1931	closer to Winterhaus weekly/monthly with interruption in winter, less frequent further inland	can be combined with density measurements; (T); (*)
159	Mass balance	Stake measurements on the outlet glacier in 50, 270, 570 and 950 m a.s.l.	31.July 1929 - 4. October 1931	Irregular (ca.weekly-monthly) during between May and October	(T); (*)
163	Mass balance	Time series of the different locations along the track to Eismitte			(F)
167	Figure stake observations ablation zone				(F)
172	Snow sweep	Discussion about how much firn loss is due to snow sweep			(C)
177	Biological and anthropological results	Characterisation about sledge dogs			(C)
199	Map of western area	Sketch			(F)

Volume 2 Seismic

This volume discusses the measurement of the ice thickness with the echolot.

Page	What	Details	Period	Resolution	Comments
3	Seismograms	At Weststation, RA062, RA082 and RA120			(F)
48	List of blasts	List of all seismic blasts between Weststation and 120km of the ice margin			(T)
51	Introduction of method	History and method of thickness measurement with seismic			(C)
73	Runtime of measurements	Observed and calculated runtime of a seismic measurement at Weststation, RA062, RA082 and RA120			(C)
86	Reflection of measurements	Observation of a reflected signal to calculate ice thickness at Weststation, RA062, RA082 and RA120			(C)
92	Additional thoughts to seismic observations	E.g. Bedrock elevation			(C)
127	Seismograms	Of pre-expedition and at Eismitte			(F)
141	Ice thickness	Results of the pre-expedition			(C)
149	Ice thickness	Results of Eismitte			(C)

Volume 3 Glaciology

This volume summarizes the glaciological observations at Eismitte and Weststation.

Page	What	Details	Period	Resolution	Comments
1	Description of local glaciers at Westcoast	Description and sketched maps of the local glaciers around the Weststation			(C)
18	Description of study area Weststation	Details about the location, the methods and the Weststation			(C)
30	Density of ice	Short list and description of the measurement of glacier ice	January 1931	At 5,5 and 12,8m depth	(T)
31	Ice temperature measurements	in the ice shaft at the Weststation, Including a correction table of the thermometers	January – June 1931	Every 0,5m down to 19,5m	One additional measurement above the surface and one at 0,23m depth to show the influence of the air on the ice temperature; (T)
39	Isotherms in the ice	Isotherms of the ice in the shaft, additionally the monthly ice temperature gradient through	January – Mai 1931		(F)
40	Monthly mean temperature gradient in the ice	Ice temperature averaged over the month and the depth profile is shown	January – June 1931		(F)
41	Thoughts about the temperature distribution of the ice sheet (C)	Connection to the seismic measurements and showing the potential of it; Righeitsmodul, elasticity module, compressibility module			(C)

53	Yearly accumulation at RA120 and RA62 (C)	Detailed description about the identification of yearly layers to average the yearly accumulation			(C)
62	General explanation about Eismitte (C)	Where, who and what was station Eismitte			(C)
79	Temperature in living space and firn at Eismitte	Temperature measurements in the living space of Eismitte as well as the temperature in the firn walls around	7. October – 8. December 1930	Irregular hourly/daily, air temperature 90cm below ceiling, firn temperature 40cm below ceiling at 10, 50 and 100cm depth	(T)
83	Air movement within the firn	Observation of air flow out of the thermometer holes in the wall when there is a cyclone	16. October 1930 – 8. April 1931		(C)
94	Firn/snow density at Eismitte	Snow and firn density measurements at Eismitte station from the surface to eventually 15m depth	September 1930 – April 1931	Daily/weekly	(T); (*)
96	Additional Firn observations	Layering, hardness, translucency, water absorbency, grain size			(C)
108	Newest snow layer observation	The newest snow layers were observed from depth, hardness, grain size, density and water equivalent at Eismitte	At 7. January, 3. February, 20. March, 25. April 1931		(T)
110	Yearly layer firn	Identification of the yearly firn layers with depth, density, hardness , grain size and divided into seasonal layers at Eismitte	1929 - 1911		(T)

121	Timeseries percental precipitation sum	Comparing Upernavik and Eismitte			(F)
123	Comparison precipitation Upernavik and water equivalent firn at Eismitte	Precipitation at Upernavik and water equivalent of firn layers at Eismitte are compared yearly and seasonal to support the identification of the seasonal layers in the ice.	1911-1931	seasonal/yearly	(T)
134	Firn density at Eismitte	Corrected firn density, different density gradient 0-7m and 7-15m			(F)
138	Firn shrinkage	Observation and correction of the firn shrinkage at Eismitte; followed by connected discussion with firn density	15. January - 6. August 1931		(C)
166	Firnstoß (Firn break)	Description of the observed breaks of a firn layer			(C)
174	Firn grain size	Observation of the grain size at Eismitte, photos, tables and interpretation			(C)
199	Firn temperature	Firn temperature in the shaft at Eismitte, method and results	3. November 1930 – 6. August 1931	Daily/weekly	(C)
264	Yearly mean temperature weather stations Greenland	Yearly mean temperature of stations around Greenland showing a warming trend	1913-1930		(T)

Volume 4.2 Meteorological Observation

This is the second volume of two about all atmospheric observations.

Page	What	Details	Period	Resolution	Comments
1	Discussion Eaststation	Discussion of the results at Eaststation Scoresby- Sund			(C)
3	Radiation	Monthly and hourly radiation at Eaststation	8. August 1930 - 17. July 1931	every 2 hours with gaps	(T)
13	Air pressure	Average daily cycle of air pressure at Eaststation and Scoresby-Sund as figure and table			(F); (T)
17	Comparison air temperature Scoresby-Sund	The monthly average and the yearly cycle is compared with other stations in Greenland, the continentality, the minima and maxima as well as the warming/cooling is investigated			(C)
38	Air temperature comparison of Scoresby-Sund and Eaststation	The yearly and daily cycle as well as continentality, warming and cooling is compared			(C)
57	Humidity Eaststation	Supersaturation and daily cycle of humidity at Eaststation			(C)
58	Wind Eaststation	Wind direction and frequency of wind speed at Scoresby-Sund and Eaststation			(C)
65	Cloudiness	Cloudiness and foggy days at Scoresby-Sund and Eaststation			(C)

69	Results of soundings	Discussion of air temperature, humidity, wind profiles at Eaststation and Scoresby-Sund			(C)
87	Discussion Eismitte	Discussion of the sounding results at Eismitte as well as wind, humidity, air and snow temperature, radiation. The data is published in volume 4.1.			(C)
124	Additions to data volume 4.1	Daily average as well as average hourly per month air temperature, daily average humidity and average hourly per month air pressure			(C)
130	Contextualize observation to synoptic	The observation at Eismitte are put into context with the large scale synoptic situation			(C)
135	Discussion of observation RA200	The observation at 200km from the ice margin are set discussed.			(C)
139	Air temperature Weststation	Detailed analysis of the air temperature Weststation (daily cycle, temperature range)			(C)
148	Air temperature Kamarujuk station	Detailed analysis of the air temperature of the weather station at the coast in Kamarujuk fjord (daily cycle, temperature range)			(C)

152	Air temperature Uummannaq station	Detailed analysis of the air temperature of the weather station in Uummannaq (daily cycle, temperature range)			(C)
155	Air temperature Westcoast	Air temperature, föhn, number of days above freezing, long-term average			(C)
186	Air pressure Westcoast	Yearly, daily cycle of air pressure at Weststation and Uummannaq station			(C)
192	Humidity Westcoast	Comparison West- and Eastcoast, saturation, daily cycle			(C)
196	Wind Westcoast	Frequency of wind direction at Weststation and Uummannaq station, wind speed, high altitude winds			(C)
208	Cloudiness Westcoast	Number of cloudy and cloud free days, average cloudiness, daily cycle, cloud types			(C)
219	Precipitation Westcoast	Number of days with precipitation, amount			(C)
222	Typical Weather	Description of weather patterns typical for a period			(C)
249	Meteorological observation Kamarujuk fjord	Introduction to the weather observation at the coastline, including air temperature and humidity July 1930-September 1931			(C); (*)

274	Weather change over Greenland	Effect of Greenland on the weather patterns, discussion if the expedition set up can answer the question, detailed analysis about the development of the weather pattern			(C)
326	Additions to data volume 4.1	Additional data for Weststation and Uummannaq, correction of humidity, air pressure, wind velocity			(C)
329	Density Measurements	Snow density measurements along the transect to Eismitte	July, August 1930 and June/July 1931	Differs between surface layer and 1.8m depth	Includes also altitudes of RA locations; (T); (*)
337	Discussion density observation	Influence of the season, snow depth, local differences			(C)
342	Seasonal layers	Depth and thickness of seasonal layers along the transect			(C)
346	Glaciological observation RA200	Drill, location, results of layers, grain size, densities of firn			(C)
356	Glacier Jakobshavn	Velocity and glacier front of glacier Jakobshavn			(C)
363	Summary meteorological Results	Summary of radiation, statistical analysis of air temperature, weather patterns over Greenland, vertical profiles			(C)

Volume 4.1Meteorological Observation

This is the first of two volume about all atmospheric observations.

Page	What	Details	Period	Resolution	Comments
2	Meteorological measurements Eaststation	Air pressure, air temperature, extreme temperatures, humidity, wind, clouds	8.-14. August 1930, 1. October 1930 - 16. July 1931	at 0800, 1400, 2100 local time	(T)
24	Barographic observation	Timeseries of Air pressure	30. September 1930 - 17. July 1931	Every two hours	(F)
33	Balloon/Kite sounding at Eaststation	Height, air pressure, air temperature, humidity, wind	9. August 1930 - 16. July 1931	Daily/weekly	(T)
65	High altitude winds Eaststation	Wind direction and speed at different altitudes	8. August 1930 - 14. July 1931	Every 200m, Irregular between weekly/monthly	(T)
116	Radiation	Net radiation at Eaststation and Scoresby - Sund	August 1930 - July 1931		(F)
124	Instruments/Methods	Introduction to used methods and instruments at Eaststation			(C)
176	Altitude Eismitte	Estimate of the altitude at Eismitte based on pressure gradient			(C)
191	Station Eismitte	Introduction to tasks, set-up and location of Eismitte station; schedule			(C)
212	Meteorological measurements Eismitte	Air pressure, air temperature, extreme temperatures, humidity, wind, clouds	31. July 1930 - 6. August 1931	at 0800, 1400, 2100 local time	(T)
237	Air pressure	Air pressure at Eismitte	6. August 1930 - 6. August 1931	hourly	(T)
250	Air temperature	Air temperature at Eismitte	1. August 1930 - 6. August 1931	hourly	(T)

263	Humidity	Humidity at Eismitte	1. August 1930 - 5. August 1931	hourly	(T)
271	Radiation	Radiation at Eismitte	5. August 1930 - 6. August 1931	hourly	gap during polar night; (T)
280	Sunshine period	Sunshine period at Eismitte	1. August 1930 - 5. August 1931	hourly	(T)
289	Air pressure	Air pressure at Eismitte	6. August 1930 - 6. August 1931		(F)
300	Air temperature and humidity	Air temperature and humidity at Eismitte	31. July 1930 - 6. August 1931		(F)
318	Radiation	Radiation at Eismitte	5. August 1930 - 6. August 1931		(F)
324	Instruments used at Eismitte	Instruments used for meteorological observations at Eismitte, including a error discussion and correction calculations			(C)
357	Introduction balloon sounding	Introduction of the instruments and method of the balloon soundings			(C)
367	Balloon soundings for station Eismitte	Height, air pressure, air temperature, humidity	15.August 1930 -13. July 1931		(T); (*)
375	Temperature gradient	Temperature gradient of the different height layers for the different balloon soundings			(T)
377	Winds Eismitte	Distribution of observed wind direction for 1930 and 1931			(T)

380	Clouds	Cloud observation Eismitte	1. September 1930 - 1. August 1931		(T)
388	Meteorological measurements Uummannaq	Air pressure, air temperature, humidity, wind, clouds, precipitation	20.June 1930 - 30. September 1931	at 0800, 1400, 2100 local time	(T)
404	Station Weststation	Set-up, instruments and methods at Weststation			(C)
412	Meteorological measurements Weststation	Air pressure, air temperature, humidity, wind, clouds; followed by the recorded stripes, first Scheideck then Winterhaus	10. August - 31. October 1930 (Scheideck); 1. November 1930 - 19. September 1931 (Winter haus)	0800, 1400, 2100 local time	(T)
448	Air pressure	Air pressure at Weststation	11. November 1930 -19. September 1931	Every two hours	(T)
453	Air temperature	Air temperature at Weststation	6.August 1930 - 18. September 1931	Every two hours	(T); (*)
460	Comments about observations	Additional comments about the observation at Weststation			(C)
466	Sounding	Kite soundings at Weststation for wind observation	17. September 1930 - 21. July 1931	Irregular	(T)
471	sun position	Day and time of sun position and intensity	5. October 1930 - 18. July 1931	Irregular	(T)
472	Sunshine period	Sunshine period at Weststation	1. August 1930 - 18. September 1931	hourly	(T)

492	Intensity of sun radiation	Intensity of the sun and sky radiation with Robitzsch in 10^-2 gcal/cm^2 min	10. August 1930 - 19. September 1931	hourly	(T)
501	Results of radiation observation	Summed up result of radiation observation, including sun radiation on a perpendicular surface and normed intensities			(C)
513	Temperature snow surface	Temperature of boundary layer air - snow	13. January - 12. February 1931	Daily	(T)
518	Air pressure differences	Differences between Scheideck and Uummannaq in air pressure and air temperature	20. June 1930 - 30. September 1931	Every 5th day	(T)
526	Simulatneous observations Uummannaq and Scheideck	Temperature, wind direction and speed	3.- 12. November 1930; 13. June - 9. August 1931	at 0800, 1400, 2100 local time in Winter, irregular in summer	(T)
531	Tidal height Kamarujuk Fjord	Method and Observaiton about the tidal height in the Kamarujuk fjord, additionally correction and influence air pressure	28. June - 12 July 1930	daily	(C)

Volume 5 Geodesy

This volume includes the geographical localization, gravitation measurements and wind speed results of Weststation.

Page	What	Details	Period	Resolution	Comments
1	geographic localization	Introduction on method and instruments used to determine the geographic location			(C)
30	coordinates	coordinates of area around Weststation and the transect to Eismitte			(T)
31	coordinates of Eismitte	Discussion on the accuracy of the coordinates of Eismitte			(C)
33	Gravity measurement with pendulums	Introduction and on method and instruments used to measure gravitation including accuracy and error discussion			(C)
73	Wind speed Weststation	Results and discussion of the wind speed at Weststation including an average daily cycle of minima and maxima	10. August 1930 - 17. September 1931	hourly	(T)

Volume 6 Anthropology and Zoology

This volume describes the results of anthropological studies and zoology.

Page	What	Details	Period	Resolution	Comments
2	Local community Scoresby-Sund	Observation of the local population in Scoresby-Sund			(C)
85	Remains of population	Observation of the found remains in the area of Scoresby-Sund			(C)
175	Fauna groups	The different fauna groups found in Scoresby-Sund, ice-region, land-region, bird cliff, hot springs			(C)
180	vulnerability of wildlife	thoughts about how the new hunting techniques can endanger polar bear, fox and seals			(C)
183	ornithological observations	new observations of birds in Greenland			(C)
185	parasitic worms	observations of worms in Scoresby-Sund area			(C)
190	bacteria in polar wildlife	bacteria in the gastrointestinal tract of different polar animals			(C)

Volume 7 Summary

This volume summarizes the meteorological and glaciological observations of the expedition.

Page	What	Details	Period	Resolution	Comments
4	Wind	Summary about the wind observation and results			(C)
6	Air temperature	Summary about the air temperature observation and results			(C)
11	Air pressure	Summary about the air pressure observation and results			(C)
16	Radiation	Summary about the radiation observation and results			(C)
18	Humidity	Summary about the humidity observation and results			(C)
20	Clouds	Main message that cumulus clouds are not observed inland.			(C)
20	Air temperature and air pressure	Discussion about the connection of air temperature and air pressure observations			(C)
27	Glacial anticyclone	Discussion about stationary and moving anticyclones, lowest air masses and their direction			Mentioning sounding experiments not possible above 400m; (C)
31	Weather	Summary about typical weather patterns, Greenland's influence on the weather in Europe, polar air outbreaks			(C)
35	Air traffic to Greenland	On the potential of air traffic to Greenland			(C)

36	Mass balance	Accumulation, ablation, transport of ice/firn grains			(C)
40	Randgebiet	Characteristics of the ice margin with snowline, crevasses, melt rates			(C)
41	Firn density	The density at different depths and seasonal difference			(C)
44	Ice temperature	Ice temperature and thoughts about the minimal ice thickness calculated from the temperature gradient			(C)
50	Ice thickness	Summary about the seismic methods and results			(C)
72	Glacier fronts Uummannaq	Reference to Volume 3			(C)
